# The Association between Dietary Inflammatory Potential and Urologic Cancers: True Association or Bias?

**DOI:** 10.1016/j.advnut.2023.10.005

**Published:** 2023-11-03

**Authors:** Yiwen Zhang, Edward Giovannucci

**Affiliations:** Department of Nutrition, Harvard T. H. Chan School of Public Health, Boston, MA, United States; Department of Nutrition, Harvard T. H. Chan School of Public Health, Boston, MA, United States; Department of Epidemiology, Harvard T. H. Chan School of Public Health, Boston, MA, United States

In the current issue, Dai et al. [1] presents a meta-analysis of 23 studies and 557,576 participants that found that a proinflammatory diet was associated with higher risk of prostate and kidney cancer [1]. This finding aligns with a meta-analysis performed in 2018 and seems to support the role of diet-induced inflammation in the risk of these cancers [2]. The authors should be commended for their comprehensive work in summarizing the literature. Three important features of the results merit consideration. These include the different findings by study design (case-control compared with cohort), differences whether relying on common-effect models or random-effect models, and differences in inflammatory diet assessment methods, the dietary inflammatory index (DII) and the empirical dietary inflammatory pattern (EDIP). Specifically, positive associations were largely limited to case-control studies, for random-effect models, and for the DII.

Regarding study design, compared to case-control studies, higher reliability is generally bestowed upon cohort studies, where diet is measured before occurrence of the outcome and is therefore less likely to be influenced by the outcome. For example, the World Cancer Research Fund/American Institute of Cancer Research essentially only considers cohort studies and randomized control trials to make robust conclusions about diet and cancer [3]. Both cohort and case-control studies relied on self-reports of one’s diet, which is prone to measurement error. Yet, in cohort studies, the assessment occurs years or decades before the cancer diagnosis, so measurement error is presumably nondifferential or random between the ensuing cases and noncases. Thus, any dietary reporting bias in cohort studies tends to attenuate any real association but will generally not generate false associations. For case-control studies, as the study participant or investigator is not blinded to their disease status, various biases, particularly recall bias, may arise and potentially produce spurious associations. In fact, this tendency for bias in case-control studies has been empirically demonstrated [4]. Further, many case-control studies used patients hospitalized for other reasons to sample the underlying (“control”) population, which could cause selection bias. Thus, the fact that an elevated prostate cancer risk was seen among case-control studies (relative risk [RR]: 1.75; 95% confidence interval [CI]; 1.34, 2.28) but not among cohort studies (RR: 1.02; 95% CI: 0.96, 1.08) should raise suspicion.

A second difference in the results was that the estimates differed depending on whether the authors used the common-effect model (e.g., for prostate cancer; RR: 1.04; 95% CI: 1.01, 1.07) or the random-effect model (RR: 1.52; 95% CI: 1.23, 1.88). The common-effect model assumes 1 true underlying association common to all studies in the meta-analysis, and the observed difference arises solely from within-study sampling variability; in contrast, the random-effect model allows for the possibility that the true effect size might vary between studies beyond sampling variability, acknowledging heterogeneity in the true underlying effects. Although it is often assumed that the random-effect estimate is more conservative, the random-effect estimate tends to give more weight to smaller studies with more extreme findings. In this meta-analysis for prostate cancer, the studies with stronger associations were largely the smaller case-control studies. Not only are these more prone to recall and selection bias, as noted above, these results are also susceptible to publication bias, as demonstrated by the authors. The studies with the strongest associations had a small number of cases (e.g., 50, 50, 60, and 72), while the largest cohort study had 5929 cases. Thus, the common-effect model, which gives more weight to the large cohort studies as opposed to smaller case-control studies, may be considered more reliable in this case.

A third key difference was that associations were limited to studies that used the DII rather than the EDIP. Could this difference explain the divergent results? Briefly, to formulate the DII, qualifying articles from the literature were scored based on the effect of 45 food parameters on 6 inflammatory biomarkers: IL-1β, IL-4, IL-6, IL-10, TNF-α, and C-reactive protein [5]. The food parameters included both foods (e.g., alcohol, garlic, and tea) and nutrients and phytochemicals (e.g., β-carotene, magnesium, and flavonoids). Nutrient supplements counted toward nutrient intake. The EDIP was developed among United States health professional populations [6]. Thirty-nine predefined food groups were entered into reduced rank regression models, followed by stepwise linear regression to identify a dietary pattern most predictive of 3 inflammatory markers: IL-6, C-reactive protein, and TNFαR2. Although the DII and EDIP may each have specific merits and limitations, it is unlikely that they are substantially different in predicting inflammation, at least in United States populations [7]. In the meta-analysis, for prostate cancer, studies based on the EDIP were null for both the common-effect and random-effect models (RR: 0.99; 95% CI: 0.95, 1.02), whereas for the DII, the results were positive but much stronger for the random-effect model (RR: 1.67; 95% CI: 1.32, 2.11) than for the common-effect model (RR: 1.14; 95% CI: 1.09, 1.19). Thus, the main difference between the DII and the EDIP is that the EDIP was used exclusively in large cohort studies and the DII tended to be used in small case-control studies.

Another potential contributor to inconsistent results across studies is that prostate cancer is a heterogeneous disease, and the aggressive forms could be a more clinically important endpoint. Aggressive prostate cancer has been shown to be more associated with modifiable lifestyle factors, including diet. Different prostate specific antigen screening policies might also complicate the story. In the United States and Canada, where prostate specific antigen screening was more prevalent, the overall prostate cancer cases were most likely to be indolent, while in countries where screening was less common, the prostate cancer cases diagnosed could be more advanced. However, cohort studies conducted in the United States (comprising 99% of all the cases from the cohorts) examined aggressive or high-risk prostate cancer separately and did not show clear associations for it [[8], [9], [10]]. Thus, the composition of prostate cancer cases is unlikely to explain the heterogeneity in results.

In conclusion, the 3 apparent differences in the results reduce to whether emphasis is based on smaller, case-control studies or larger, cohort studies. The studies that support an association are more prone to recall, selection, and publication bias, suggesting that conclusions of a positive association should be tempered. More large-scale cohort studies on inflammatory diet and urological cancer risk and carefully conducted meta-analysis are needed. For prostate cancer, careful attention should be given to aggressive forms of the disease.

## Author contributions

YZ: Conceptualization, Writing - original draft.

EG: Conceptualization, Writing - review & editing.

## Conflict of interest

The authors report no conflicts of interest.

## Funding

The authors reported no funding received for this study.

## Declaration of interests

The authors declare that they have no known competing financial interests or personal relationships that could have appeared to influence the work reported in this paper.
